# Green Preparation of Straw Fiber Reinforced Hydrolyzed Soy Protein Isolate/Urea/Formaldehyde Composites for Biocomposite Flower Pots Application

**DOI:** 10.3390/ma11091695

**Published:** 2018-09-12

**Authors:** Enhui Sun, Guangfu Liao, Qian Zhang, Ping Qu, Guofeng Wu, Yueding Xu, Cheng Yong, Hongying Huang

**Affiliations:** 1 Circular Agriculture Research Center, Jiangsu Academy of Agricultural Science, Nanjing 210014, China; enhsun@126.com (E.S.); qupinghappy@163.com (P.Q.); guofengwu@126.com (G.W.); yueding_xu@163.com (Y.X.); yongcheng0520@hotmail.com (C.Y.); 2 Jiangsu Agricultural Waste Treatment and Recycle Engineering Research Center, Nanjing 210014, China; 3 School of Materials Science and Engineering, PCFM Lab, Sun Yat-sen University, Guangzhou 510275, China; lgfhubu@163.com; 4 Dynea (Nanjing) Chemistry Co., Ltd., Nanjing 210014, China; zhangqianlulu@126.com

**Keywords:** biodegradable polymers, mechanical property, thermal property, degradation property, biocomposite flower pots application

## Abstract

The effects of soil burial on the biodegradation of biocomposite flower pots (BFP) made from straw fiber (SF) and hydrolyzed soy protein isolate/urea/formaldehyde (HSPI/U/F) copolymer resin were studied in detail. The microstructure, crystallinity, functional groups, mechanical, degradation and thermal property of the prepared SF with HSPI/U/F copolymer resin have been studied, and the degradation mechanism was also elucidated. XRD results showed that the bond breakage between SF and HSPI/U/F copolymer resin induced a decrease in relative degradation-resistant crystal structures. FTIR spectra showed that the methylolated HSPI units could form a cross-linking network with U/F and SF. The BFP degradation after soil burial was mainly attributed to the effects of microorganisms. The degradation products were environmentally friendly, because they were degradable and could fertilize the soil. In addition, the U/F adhesives were slightly degraded by the microorganisms due to the HSPI in the pots. The TG and DSC results showed that the molecular motion of the BFP matrix could be restricted by the degradation action and the content of HSPI, resulting in decreased crystallization enthalpy and showing good thermal property. The tensile strength of different reinforced samples was not significantly reduced in comparison to U/F resin, and still kept good mechanical performance. Thus, the prepared SF reinforced HSPI/U/F copolymer resins could have good potential for use in the field of biodegradable flower pots because of their good thermal property, mechanical property, biodegradability, and relatively low cost.

## 1. Introduction

During recent decades, one of the most widely discussed topics in the green industry, which is propagated by consumers exhibiting greater degrees of environmental awareness, is environmental sustainability [1,2,3,4,5,6,7,8,9,10]. Commercial greenhouse and nursery industries often produce crops in plastic pots of varying sizes and shapes, depending on the crop and target market. Plastic pots serve as consumer packaging, plant transportation, a propagation and production receptacle, and sometimes a marketing mechanism. Nearly every floral crop and many nursery crops are grown in plastic pots [11,12].

Due to increasing concerns about the amount of landfill waste generated by plastic and pseudo-environmental consciousness related to high fuel prices, the floriculture industry has seen a rise in the use of biodegradable, compostable or bio-resin plastics, which are often considered “green” or “sustainable” products [13,14,15]. These pots are derived from renewable raw materials (e.g., corn or wheat starch, and rice hulls), cellulose, soy protein, and lactic acid. Therefore, they are often labeled as compostable because they are broken down by naturally occurring microorganisms into carbon dioxide, water, and biomass when composted or discarded [16]. These biodegradable pots can be planted directly into soil or composted and will eventually be broken down by microorganisms [17]. Environmental problems caused by petroleum-based plastics have led to an interest in biodegradable polymer (bioplastic) alternatives, but the effort to evaluate the horticultural pots made from these materials is rarely reported.

Recently, a study utilizing and comparing hypothetical conjoint analysis and non-hypothetical experimental auctions has been conducted to determine floral customers’ willingness to pay for biodegradable plant containers. The results show that participants are willing to pay a premium price for biodegradable pots; however, the prices vary for different types of pots [18]. Various fiber products can also be sourced from manure. Fiber boards and decking planks are examples of products that use equal parts dried manure and recycled corrugated products. An extension of the fiber boards and decking is the manufacturing of building materials from recycled products. Additionally, cow pots formed from manure and recycled products are marketed for use as biodegradable flower pots and pots used in the landscape and environment horticulture industry [19].

Among them, urea/formaldehyde (U/F) adhesives is the major resin and has been recently used in the wood industry because of its high bonding strength and its lesser cost than other adhesives [20]. Its application field involves the manufacturing of plywood [21], particleboard and medium-density fiberboard [22]. U/F adhesive possesses some advantages such as fast curing, good performance in panel, water solubility, and low price. It is very appropriate if these advantages are used in the production aspect of biocomposite flower pots (BFPs). However, with increasing concern about environmental pollutants produced from the formaldehyde of the U/F resin, researchers have focused on environmentally friendly polymers for reducing the amount of formaldehyde. Lots of effort has been taken to reduce formadehyde emissions by adding compounds into U/F, including core flour [23,24], glycolysis products [25], scallop shell nanoparticles [26], nano SiO_2_ [27,28,29], nanocrystalline cellulose [30], and corn flour [31].

In our past works, we found that use hydrolyzed soy protein isolate (HSPI) to partially substitute U and synthesize the HSPI modified U/F adhesives by copolymerization. The results show that the degradation of HSPI/U/F adhesive is apparently higher than that of unmodified U/F in biologically active soil [32,33]. Furthermore, the formaldehyde emission is dramatically reduced. To the best of our knowledge, the work described here represents the first attempt to make plant pots from straw fiber (SF) and a hydrolyzed soy protein isolate/urea/formaldehyde (HSPI/U/F) copolymer resin. Furthermore, flower pots with SF and HSPI/U/F adhesive are not widely produced in the world. There are few publications on the biodegradation of buried SF and HSPI/U/F copolymer resin [34,35]. Thus, the main objective of this research was to determine the mechanical and degradation property of the BFPs in a soil composting medium. In this work, we prepared novel biodegradable biocomposite flower pots (BFP) made from straw fiber (SF) and hydrolyzed soy protein isolate/urea/formaldehyde (HSPI/U/F) copolymer resin. The prepared SF reinforced HSPI/U/F copolymer resins could have a high potential for use in the field of biodegradable flower pots because of their good thermal property, mechanical property, biodegradability, and relatively low cost. We can imagine that there is such a flower pot, which is low cost and very durable under normal use; if it is damaged, we just need to bury it in the soil, and it can be degraded by microorganisms within a few months. Thus, we predict that BFP has considerable application value.

## 2. Materials and Methods

### 2.1. Materials

**Figure 1:**
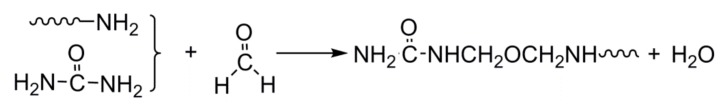
The synthesis process of copolymer resin with hydrolyzed soy protein isolate/urea/formaldehyde.

Straw Fiber (SF) feedstock was collected from an experimental station at the Jiangsu Academy of Agricultural Sciences in Nanjing, China, in August 2013. The SFs were ground to a size of 40–60 mesh. The moisture content of the prepared powder was approximately 12% after drying in ambient conditions. The hydrolyzed soy protein isolate/urea/formaldehyde copolymer resin (HSPI/U/F) was synthesized according to previous reports [36]. The synthesis process of HSPI/U/F is shown in Figure 1. The resins with 6%, 9%, 18% and 25% urea were named as HSPI/U/F-I, HSPI/U/F-II, HSPI/U/F-III, HSPI/U/F-IV, respectively. Ammonium chloride (NH_4_Cl, 20 wt.% solution) was used as a curing catalyst at a concentration of 0.01 w/w of HSPI/U/F resin.

### 2.2. Biocomposite Flower Pot (BFP) Fabrication

Typical HSPI/U/F (I-IV) resin (3500 g) was mixed with the catalyst (NH_4_Cl, 20 wt. % solution) and then poured on straw fiber (SF) power (5000 g). Next, paraffin wax (200 g) was added as a releasing agent and then the mixture was blended in a 10-L high-speed mixer (SHR-10A, China) equipped with a 2.2 kW motor at 60 °C for 20 min. The semi-finished materials, with a moisture content of 14%, were placed in a hot-press machine with nine-cavity molds at 120 °C under 13 MPa pressure for 85 s. Note, there are four types of biocomposite flower pots with 6%, 9%, 18% and 25% urea, which were named BFP-I, BFP-II, BFP-III and BFP-IV, respectively.

### 2.3. Soil Culture Experiment

Biodegradation of the BFP samples were examined using a soil burial test. The mechanical property following biodegradation was evaluated using six sample dimensions, as required by the relevant testing standards. The burial test was carried out in an enriched nutritive substrate and the specimens were buried in the compost soil in a random pattern. All film samples (dimensions 50 × 50 × 2 mm) were buried at a depth of 10 cm below the soil surface. The moisture content was maintained at 65% in order to ensure aerobic degradation [37,38]. Over the 24-month experimental period, the temperature ranged from 10–25 °C and the air humidity was 25–65%. The physicochemical property of the samples was also determined.

### 2.4. Mechanical Property and Weight Loss Tests after Biodegradation

The BFP films measured 80 mm length, 2 mm thickness and 10 mm width. The tensile strength of each film was measured before the field trial using testing instrument (HY-0580, Shanghai Hengyi Precision Instrument, Shanghai, China) according to standard protocols for measuring tension and elongation of elastic fabrics (ASTM D4964-64). Tests were conducted at a crosshead speed of 5 mm/min. Each value reported here is the average of at least six tests.

At different time points, all samples were collected from the soil, gently cleaned by rinsing with distilled water and brushing, dried to a constant weight at 105 °C, and weighted. Weight loss of the samples was used to determine the degradation rate during the soil burial test. The weight loss percentage of samples was calculated using the following equation:

Weight Loss (%) = [(W − W_0_)/W] × 100% (1)

where W_0_ is the specimen weight after degradation and W is the specimen weight before degradation. The weight loss percentage was taken from the average of three samples.

### 2.5. Characterization

Fourier transform infrared spectroscopy (FTIR) was used to obtain spectra of the BFP polymer with an infrared spectrometric analyzer (Tensor 27, Bruker, Karlsruhe, Germany) using KBr pellets in the spectral range of 4000–5000 cm^−1^. Scans (*n* = 32) were accumulated at a resolution of 4 cm^−1^. The differential scanning calorimetry (DSC) curve of the samples was obtained using a Mettler Toledo DSC apparatus with a refrigerated cooling system (DSC 823e, Schwerzenbach, Switzerland) and nitrogen as the purge gas. Thermogravimetric analysis (TGA) was used to assess whether interactions between the two organic phases influenced the thermal degradation of the composites. Samples were placed in alumina crucibles and tested with a Perkin-Elmer TGA-7 thermal gravimetric analyzer (SII-7200, Hitachi Limited, Tokyo, Japan) at a heating rate of 20 °C/min from 20 to 800 °C under nitrogen flow. In addition, the prepared flower pots were subjected to X-ray diffraction (XRD, Shimadzu diffractrometer model DX-2000, Darmstadt, Germany) operating at 40 kV voltage and 40 mA using Cu Kα radiation. The patterns were obtained within a 5 to 40° 2θ angular interval at a 0.05° step and scan speed of 2°/min. The degree of crystallinity was calculated according to the ratio of the intensity differences in the peak positions. The morphology of the samples after tensile strength testing was investigated using SEM (FEI QUANTA 200, Hillsboro, OR, USA).

## 3. Results and Discussion

### 3.1. Chemical Structure

**Figure 2:**
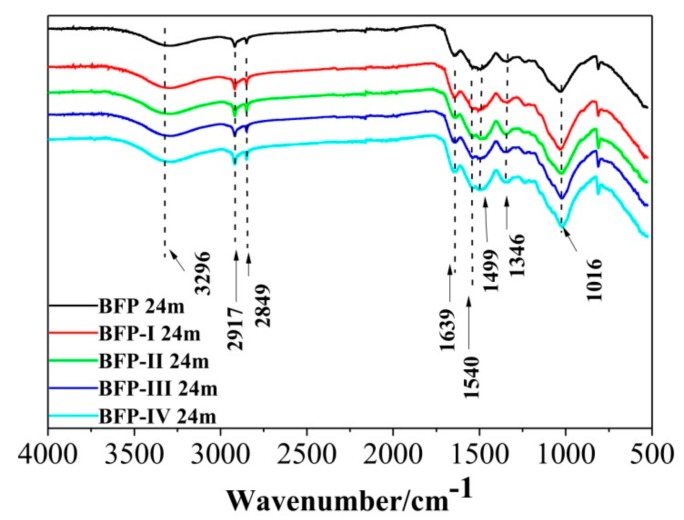
FTIR spectra of BFP, BFP-I, BFP-II, BFP-III, and BFP-IV composites.

Note, there are four types of biocomposite flower pots with 6%, 9%, 18% and 25% urea, which were named BFP-I, BFP-II, BFP-III and BFP-IV, respectively. FTIR spectra of the BFP, BFP-I, BFP-II, BFP-III, and BFP-IV composites are presented in Figure 2. The three most important characteristic peaks of HSPI are C=O stretching vibration, N-H bending vibration, and C-N stretching. A high similarity is noticed when compared to the spectra of these BFP composites (Figure 2). A strong absorption peak appeared at approximately 3296 cm^−1^, which was assigned to the free and bounded O-H and N-H functional groups. The O-H and N-H groups in the HSPI structure form inter-and intra-molecular hydrogen bonding with the C=O moiety of the amino acids (peptide and carboxyl groups). Furthermore, hydrogen bonding between protein molecules can usually increase the BFP bonding strength. The characteristic C-H symmetrical stretching vibration band of the CH_3_ and CH_2_ groups of saturated structures (peaks at 2917 cm^−1^ and 2849 cm^−1^) became stronger. The absorption peaks at 1639 cm^−1^ belonged to the stretching vibration band of the -C=O functional group and the absorption bands in the 1540 cm^−1^ to 1499 cm^−1^ region were assigned to the bending vibration band of the N-H functional group. The characteristic absorption peak of the stretching vibration band of the C-N group appears at 1346 cm^−1^. The absorption band at 1016 cm^−1^ is attributed to C-O stretching vibrations. These results indicate that amino and carboxyl groups of HSPI are able to copolymerize with U/F, and the methylolated HSPI units can form a cross-linking network with U/F and SF.

### 3.2. Morphology and Mechanical Tensile Property

**Figure 3:**
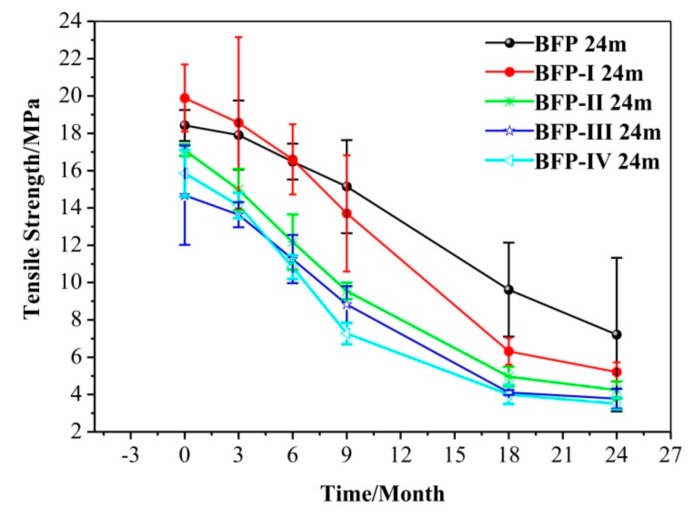
Effect of HSPI content on the tensile property of the BFP-I, BFP-II, BFP-III, and BFP-IV and BFP composites.

Figure 3 shows the effect of hydrolyzed soy protein isolate (HSPI) content on the tensile property of the BFP, BFP-I, BFP-II, BFP-III, and BFP-IV composites. The tensile strength (TS) of BFP-I composites increased from 18.15 to 19.89 MPa with a 6% HSPI content; however, BFP-II, BFP-III, and BFP-IV decreased from 18.42 to 17.09, 14.68 and 15.85 MPa, respectively. This result indicated that the TS can be improved by HSPI at an appropriate additive quantity, this is mainly attributed to the fact that HSPI can copolymerize with urea and formaldehyde to form a network structure. The HSPI content at which the value of TS plateaus could be considered the critical interfacial concentration of the BFP, which is the minimum value of interfacial saturation for a modified enhancer in the dispersed phase. This may be attributed to enhanced interfacial adhesion between the polymer matrix and SF filler by adding HSPI which is consistent with the FTIR results of all BFP composites.

For these BFP composites, the TS gradually decreased as buried soil degradation time progressed; however, there are small differences between BFP-II, BFP-III, and BFP-IV. For example, the TS of BFP-I was always greater than that of BFP in the 6 months prior to treatment, while the TS was significantly lower than BFP after 24 months where it decreased sharply from 7.24 to 5.19 MPa. Straw fiber plays an important role in the mechanical property of polymers [39,40]. During the curing of HSPI/U/F adhesives in the hot press, some of the faction groups react with various lignocellulose chemical constituents. The strength of BFP is associated with the hydrogen bond and ester bond formed during the chemical cross-linking reaction. However, microbial interactions of specific soil microorganisms, such as Aspergillus, Trichoderma, Phanerochaete and Coprinus, are considered an effective decomposition mechanism for buried BFPs [41]. They produce enzymes that can degrade cellulose, hemicelluloses and lignin [42], which can destroy the three-dimensional structure between the HSPI/U/F and SF structure. Moreover, the final BPF degradation products are CO_2_, H_2_O, and microbially produced protein, which do not pollute the environment.

**Figure 4:**
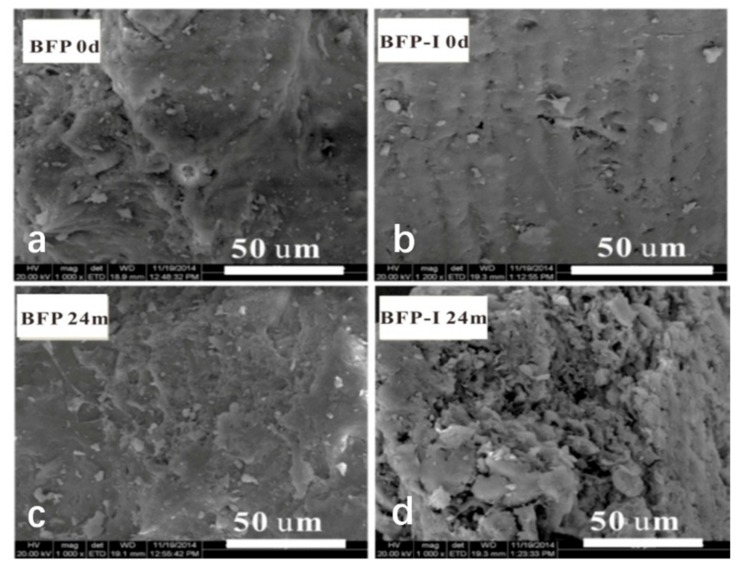
Surface morphology of the BFP and BFP-I composites. Note: (**a**) BFP untreated; (**b**) BFP-I untreated; (**c**) BFP treated 24 months of degradation; (**d**) BFP-I treated 24 months of degradation.

SEM micrographs taken from the fracture surface of two composites (BFP and BFP-I; Figure 4) showed that the surface of BFP (Figure 4a) and BFP-I (Figure 4b) samples are smooth and dense, with small holes. These microscopic structures endowed them with good mechanical strength. Many holes with different shapes were formed in the two composites after 24 months of degradation. Several broken fibers also appeared on the surface of the BFP composite. The SEM images clearly demonstrated that good mechanical property could be maintained (Figure 4c). We also observed that the interface bonding between the SF and U/F resins was better than that of the SF with HSPI/U/F resins. A greater amount of decay was observed in BFP-I after degradation. Digestion of the exposed fiber bundle began and rice straw fibers were further destroyed, resulting in many deep grooves that can clearly be seen on the surface (Figure 4d). Many fiber bundles and microfibers were exposed, and the specific surface area of the SF particles was enlarged, resulting in better contact with hydrolytic and microbial activities. In the present study, due to microbial action and hydrolysis, the BFP decomposed and broke into small fragments. Furthermore, some microorganisms penetrated the holes and digested internal components [8]. This behavior might be due to the weakened bond between SF and HSPI/U/F copolymer resin, which resulted in the reduction in the physical and mechanical property.

### 3.3. Biodegradation in Soil

**Figure 5:**
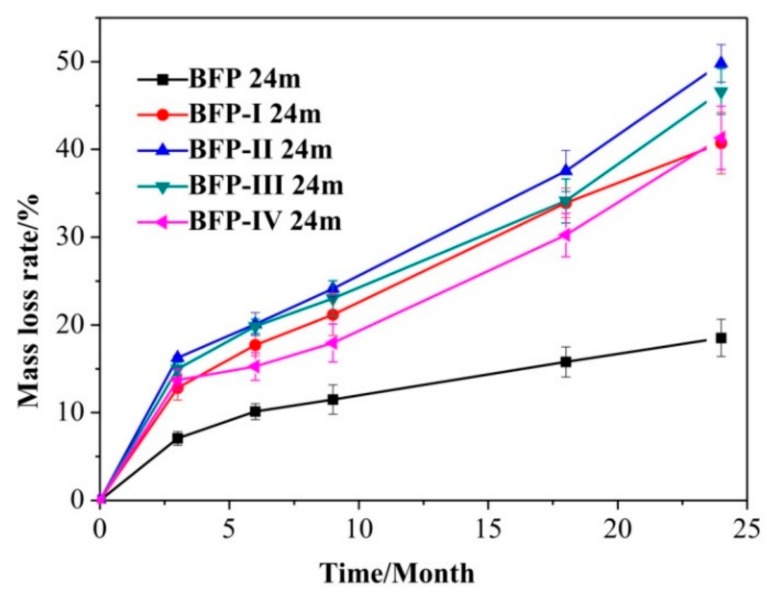
Mass loss rate of BFP, BFP-I, BFP-II, BFP-III, and BFP-IV composites. Error bars represent the standard deviation of actual test values (*n* = 3).

The variation of the mass loss rate with degradation time for BFP-I, BFP-II, BFP-III, and BFP-IV and BFP composites in soil was investigated (Figure 5). The degradability increased up to 40% as the soil burial time increased to 24 months. After 24 months of degradation, the BFP-I, II, III, and IV materials showed a higher mass loss rate than BFP and, in general, the mass loss rate increased with increasing of HSPI content, except for the BFP-IV composite. All of the BFP composites degraded rapidly within the first 3 months. This rapid degradation was mainly attributed to the composting process, which occurred in two main stages: An active composting stage and a curing period.

The peptide chain of HSPI will rupture in the process of HSPI/U/F formation, resulting in different sizes of short-chain amino acids. A large number of molecular chain amino and carboxyl groups further graft with U/F by means of condensation, thus some easily degradable segments are embedded in the three-dimensional network structure. In addition, water enters the polymer matrix of all BFP composites, which causes swelling of the fibrous material and weakens bonds. Water infiltration initiates polymer hydrolysis, leading to creation of oligomers and monomers. Consequently, microbial attack is more feasible. These results are consistent with the results of the TS analysis.

### 3.4. Thermal Property

**Figure 6:**
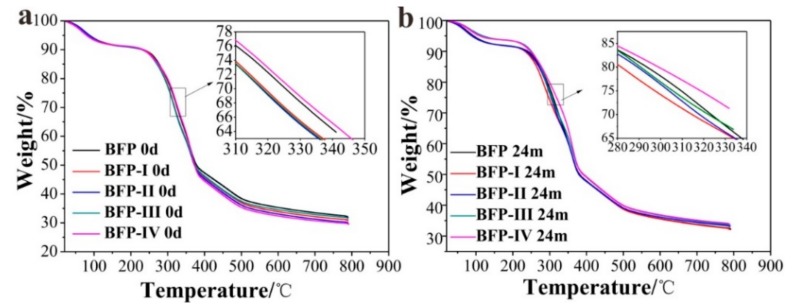
TGA curves of before (**a**) and after (**b**) degradation of BFP, BFP-I, BFP-II, BFP-III, and BFP-IV composites.

The thermal stabilities of the BFPs composites were measured by TGA under a nitrogen atmosphere. Because thermal decomposition can cause defunctionalization of HSPI, we used TGA to determine the effect of HSPI content of HSPI/U/F copolymer resin on the weight loss of the composites. The results of these analyses are summarized in Figure 6 and Table 1. The metric indexes, such as 30% decomposition temperature (T_d, 30%_) are usually used to evaluate thermal stabilities [43,44,45,46,47]. The minor weight loss observed from room temperature to 250 °C for all samples is likely due to the evaporation of adsorbed moisture. The T_d, 30%_^a^ of BFP was higher than that of BFP-I, II, III. By increasing the HSPI content of HSPI/U/F in BFPs from 6 wt.% to 25 wt.%, T_d, 30%_ in N_2_ shifted to a higher temperature (315.60, 317.25, 318.38 and 326.98 °C, respectively). However, when the content of HSPI was 25 wt.%, the T_d, 30%_ of BFP-IV composites was higher than that of pure BFP (325.42 °C and 326.98 °C, respectively). T_d, 30%_^b^ showed a similar trend. T_d, 30%_ of BFP decreased due to the copolymerization with HSPI in the copolymer resin, which can be explained by some previously reported mechanisms [33]. Thermal stability of BFP became better as HSPI content increased. The reason is the stronger interactions reformed among the exposed groups in the drying process through Van der Waals forces, intermolecular hydrogen bonds and hydrophobic interactions, thereby reducing the segmental motions and creating a barrier that delays the diffusion of heat [48].

The enhancement of the thermal stability of the BFP (I, II, III and IV) after 24 months degradation belongs to decomposition of the most stable units of methylenediurea in the HSPI/U/F, which means that the BFPs (24 m) possessed higher thermal stability than undegraded samples that can be seen from *R*_w_^c^ datas.

**Figure 7:**
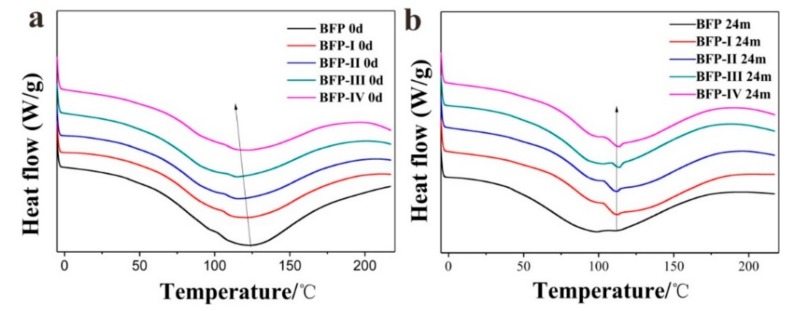
DSC curves of before (**a**) and after (**b**) degradation of BFP, BFP-I, BFP-II, BFP-III, and BFP-IV composites.

The onset temperature (*OT*), peak temperature (*PT*), and the heats of fusion (∆H_f_, enthalpy) of BFP, BFP-I, II, III, and IV composites were determined using DSC. Figure 7 shows the DSC curves for BFP, BFP-I, II, III, and IV before and after degradation samples. The thermal transition of all samples was determined with DSC by measuring the amount of energy absorbed or released. The endothermic characteristic peaks of BFP, BFP-I, II, III, and IV samples, occurring in the 50–180 °C range, are presented in Figure 7a and the results obtained from DSC are summarized in Table 2. BFP, BFP-I, II, III, and IV had peak temperatures of 121.27 °C, 115.71 °C, 114.46 °C, 114.41 °C and 115.71 °C, respectively. For all composites, the *PT* was reduced with HSPI addition, and further decreased with increasing HSPI content. These lower *PT*s, when compared to the BFP composite, indicate that the crystallization rate of the composites slow during the non-isothermal process. The decrease in *PT* could be because the modified HSPI/U/F copolymer resin was not fully cured when the BFPs were formed.

The DSC curves of BFP, BFP-I, BFP-II, BFP-III, and BFP-IV composites after sample degradation, occurring in the 40–170 °C range, are presented in Figure 7b. It is interesting to note that the curves are similar to the “shoulder peak” temperature and that the temperature shows a downward trend, which corresponds to an increasing degree of crystallinity. The *PT* of BFP, BFP-I, II, III, and IV composites shifted to higher values of 98.46 °C, 112.50 °C, 112.22 °C, 113.80 °C, and 113.62 °C, respectively. After degradation, the BFP molecular chain, with easy thermal transitions, was degraded by hydrolytic and microbial action; this resulted in reduction of the *OT* and *PT* (Table 2). Moreover, in both composites the change in ΔH_f_ became smaller because the amount of molecular chains undergoing thermal transition decreased after degradation. In addition, the molecular motion of the BFP matrix could be restricted by the degradation action and the HSPI content, resulting in decreasing crystallization enthalpy. As a result, BFP was transformed into stable and complex macromolecules under the action of microbes and their corresponding enzymes. A reduction of mechanical and physics property were also confirmed, which could be due to the partial removal of unstable molecules from the BFP composites during degradation.

### 3.5. X-ray Diffraction Analysis

**Figure 8:**
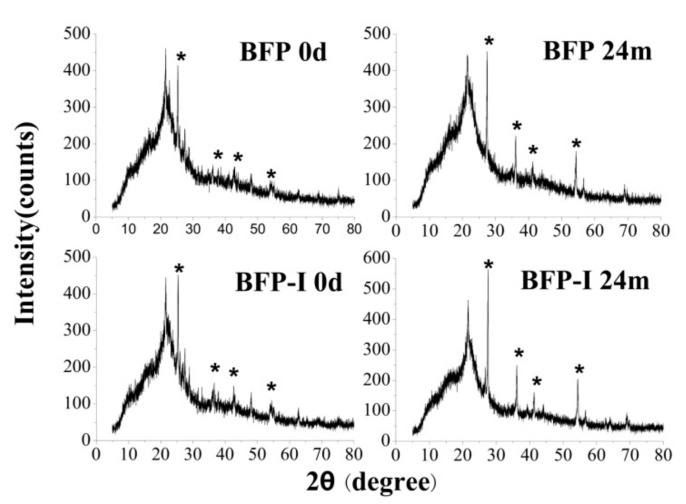
XRD curves of samples treated with different resin and at different degradation times (BFP, BFP after 24 months of degradation, BFP-I, and BFP-I after 24 months of degradation). (*) stand for characteristic peak.

The BFP crystalline structure and crystallinity were studied using X-ray diffraction (XRD; Figure 8). The strong and well-defined peaks marked with pentagrams correspond to the bleaching agent that is widely used in regenerated fiber production. This agent, titanium dioxide, is in its crystalline anatase form [49].

The crystallinity index (*C_r_I*) of the cellulose in the various BFPs was determined according to the following equation [50]:

% *C_r_I* = (*I*_002_ − *I*_am_)/*I*_002_ × 100% (2)

where *I*_002_ is the maximum diffraction intensity of the [002] crystalline lattice planes at 2*θ* of 22° to 23°, and *I*_am_ is the diffraction intensity of the amorphous cellulose, which is taken at a 2*θ* between 18° and 19°, which is where there is minimum diffraction interference from the crystalline cellulose structures.

The XRD patterns show diffraction peaks at 2*θ* around 16.9° and 21.56°, which is similar to cellulose I, corresponding to the crystal face of (110) and (200) crystal spacings of cellulose I; therefore, the addition of HSPI/U/F copolymer resin and degradation treatment did not change the crystal form, although the *C_r_I*s of all samples were significantly different from that of straw fiber [51]. The *C_r_I* values of samples, such as BFP, BFP-I, and BFP after 24 months of degradation, namely, BFP and BFP-I, were 49.45%, 41.25%, 53.09% and 50.74% (Table 3), which was calculated by the occupancy rate of the crystalline portion of the specimen [52]. Similar or decreased *C_r_I* values were observed, when compared to for BFP (49.45%). After blending with HSPI/U/F copolymer resin, the *C_r_I* values decreased to 41.25% for BFP-I, which was slightly lower than that of BFP. However, there was no significant difference between the crystallinity of SF (*P* > 0.05), demonstrating that the introduction of HSPI/U/F copolymer resin did not have any significant influence on the crystallinity of BFPs, with respect to straw fiber raw materials used in this study. A possible explanation for this could be that during blending of HSPI/U/F copolymer resin, high-temperature was required to finalize design. All of the BFPs were the same temperature in this study and this relatively low temperature might damage the crystals, thereby decreasing the *C_r_I*, which is consistent with the DSC results.

Higher *C_r_I* values for BFP (53.09%) and BFP-I (50.74%) were observed after 24 months of degradation. The increased rate of *C_r_I* for BFP was significant. Analysis of SF crystallinity is a significant criterion that is utilized to assess SF degradation during soil burial. Through microbial action, cellulose, hemicelluloses and lignin are decomposed to a certain extent. Fragments were produced because of the broken sponge-like network between SF and HSPI/U/F copolymer resin, which was clearly observed in the microscopic morphology. As a result, the bond breakage between SF and HSPI/U/F copolymer resin induces a decrease in relative degradation-resistant crystal structures. The introduced HSPI improves the biodegradable property of U/F copolymer resin. These results illustrate that the BFPs became more easily degradable in the presence of HSPI copolymer resin.

### 3.6. Degrading Mechanism

**Figure 9:**
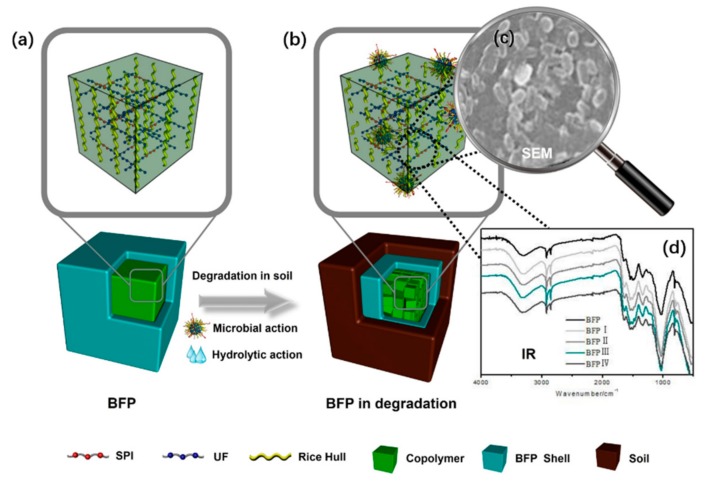
Degrading mechanism of BFP composites. (**a**) Biocomposites prepared by three-dimensional network structure. (**b**) Biodegradable composites under the condition of soil burial environment. (**c**) Abundant microorganisms adhere to the surface of biocomposites. (**d**) Changes of structural groups of degradation composites.

The degradation mechanism, degradation efficiency and degradation process of BFP are shown in Figure 9. It is found that the initial structure, chemical bond, hydrophilic and hydrophobic balance, expansion of substituents, crosslinking and chain length are very important factors to influence biodegradability. In addition, high order structures including conformation, crystallinity, morphology, and orientation also greatly affect biodegradation. Thus, the presence of HSPI can significantly improve biodegradation. The three-dimensional structure between HSPI/U/F copolymer resin and SF structure is clearly observed, showing that the bonding strength was stronger during the planting period (Figure 9a). The structural formula of BFPs is displayed in Figure 9b, and hydrolytic and microbial action can be confirmed by FTIR spectra (Figure 9d). The segment can loosen the originally dense network structure and increase the movement degree of the molecular chain. Under the action of microbial activity, it can enter the active position of the molecular chain inside the modified U/F resin and permeate to the operating point (Figure 9c). As a result, the macromolecular skeleton structure of the HSPI/U/F resin was broken, and the three-dimensional network structure was damaged, forming one-dimensional or two-dimensional fragments with low molecular weight. The fragments were mostly branched chain segments. The higher the branching degree, the more weak links in the chain structure. In addition, the degradation of BFP is fast, and is transformed into small oligomers or segments, when it is placed in a natural soil environment. This indicates that the introduced HSPI not only ensures the mechanical strength of BFP, but also accelerates the degradation of BFP.

## 4. Conclusions

The composite reinforced hydrolyzed soy protein isolate/urea/formaldehyde (HSPI/U/F) copolymer resin with waste straw fibers (SFs) is an ideal material for producing biodegradable flower pots (BFP). Among the BFP, a urea/formaldehyde cross-linked matrix reinforced with hydrolyzed soy protein isolate (HSPI) grafted straw fiber showed good thermal stability and mechanical property. The tensile strengths of different reinforced samples were not significantly reduced in comparison to U/F resin. BFPs reinforced with straw fiber and its different graft HSPI/U/F-based copolymers were found to be biodegradable, and the degradability increased up to 40% as the soil burial time increased to 24 months. As the peptide chain of HSPI ruptured in the process of HSPI/U/F formation, this resulted in different sizes of short-chain amino acids. In contrast to plastic pots, BFPs can easily decompose in soil after burial because of microorganisms, which will not pollute the environment. Furthermore, it is highly beneficial to make full use of straw fiber from agricultural residues.

## Figures and Tables

**Table 1:** Effect of HSPI content of HSPI/U/F copolymer resin on the thermal property of BFPs composites.

Sample	HSPI Content (%)	*T*_d, 30%_ (°C)		*R*_w_^c^ (wt %)	
		*T*_d, 30%_ (0 m) ^a^	*T*_d, 30%_ (24 m) ^b^	*R*_w_ (0 m) ^a^	*R*_w_ (24 m) ^b^
BFP	0	325.42	323.48	31.77	32.10
BFP-I	6	315.60	316.01	30.72	32.14
BFP-II	9	317.25	318.09	29.87	33.04
BFP-III	18	318.38	322.14	31.49	33.36
BFP-IV	25	326.98	336.04	29.46	33.81

^a^ Degradation time 0 m; ^b^ Degradation time 24 m; ^c^ Residue weight fraction at 800 °C in N_2_.

**Table 2:** Effect of HSPI and degradation time on crystallization behavior of the BFP, BFP-I, BFP-II, BFP-III, and BFP-IV materials.

Sample	HSPI Content (%)	Onset Temperature (°C)		Peak Temperature (°C)		ΔH_f_ (J/g)	
		*OT* (0 m)	*OT* (24 m)	*PT* (0 m)	*PT* (24 m)	ΔH_f_ (0 m)	ΔH_f_ (24 m)
BFP	0	49.71	37.42	121.27	98.46	184.3	141.1
BFP-I	6	53.48	76.17	115.71	112.50	143.2	136.5
BFP-II	9	52.56	75.33	114.46	112.22	139.8	137.2
BFP-III	18	50.25	53.95	114.41	113.80	132.1	126.1
BFP-IV	25	57.86	60.52	115.71	113.62	105.1	119.7

**Table 3:** Crystallinity index of different BFPs prepared by using different resins and at degradation times.

Samples	*C_r_I* (%)		Increase Rate of *C_r_I* (%)
	*C_r_I* (0 m)	*C_r_I* (24 m)	
BFP	49.45	53.09	7.36
BFP-I	41.25	50.74	23.01
